# Evaluation of the New American Joint Committee on Cancer Staging Manual 8th Edition for Perihilar Cholangiocarcinoma

**DOI:** 10.1007/s11605-019-04127-x

**Published:** 2019-02-12

**Authors:** Marcia P. Gaspersz, Stefan Buettner, Jeroen L. A. van Vugt, Jeroen de Jonge, Wojciech G. Polak, Michail Doukas, Jan N. M. Ijzermans, Bas Groot Koerkamp, François E. J. A. Willemssen

**Affiliations:** 1 Department of Surgery, Erasmus MC University Medical Center Rotterdam, ‘s Gravendijkwal 230, 3015 CE Rotterdam, The Netherlands; 2 Department of Pathology, Erasmus MC University Medical Center Rotterdam, Rotterdam, the Netherlands; 3 Department of Radiology and Nuclear Medicine, Erasmus MC University Medical Center Rotterdam, Rotterdam, the Netherlands

**Keywords:** Perihilar cholangiocarcinoma, Klatskin, TNM stage, Prognostic model

## Abstract

**Background:**

The aim was to compare the prognostic accuracy of cross-sectional imaging of the 7th and 8th editions of the American Joint Committee on Cancer(AJCC) staging system for perihilar cholangiocarcinoma(PHC).

**Methods:**

All patients with PHC between 2002 and 2014 were included. Imaging at the time of presentation was reassessed and clinical tumor–node–metastasis (cTNM) stage was determined according to the 7th and 8th editions of the AJCC staging system. Comparison of the prognostic accuracy was performed using the concordance index (c-index).

**Results:**

A total of 248 PHC patients were included;45 patients(18.1%) underwent a curative-intent resection, whereas 203 patients(81.9%) did not because they were unfit for surgery or were diagnosed with locally advanced or metastatic disease during workup. Prognostic accuracy was comparable between the 7th and 8th editions (c-index 0.57 vs 0.58). For patients who underwent a curative-intent resection, the prognostic accuracy of the 8th edition (0.67) was higher than the 7th (0.65). For patients who did not undergo a curative-intent resection, the prognostic accuracy was poor in both the 7th as the 8th editions (0.54 vs 0.57).

**Conclusion:**

The 7th and 8th editions of the AJCC staging system for PHC have comparable prognostic accuracy. Prognostic accuracy was particularly poor in unresectable patients.

## Introduction

Perihilar cholangiocarcinoma (PHC) is the most common malignancy of the bile ducts.^1^ Overall survival differs strongly between PHC patients, ranging from 12 months in palliative treatment to 40 months after curative-intent resection.^2–4^ Prognostic studies typically focus on patients undergoing curative-intent resection. However, the majority of patients with PHC have metastatic or locally advanced disease at the time of presentation.^2,5–7^

One of the most commonly used staging systems is the American Joint Committee on Cancer (AJCC) staging system. Recently, the AJCC released the AJCC 8th edition cancer staging manual, which came into effect on January 1, 2018. The 7th edition of the AJCC staging system was the first to stage PHC and distal cholangiocarcinoma separately. The new 8th edition for PHC contains four significant changes (Tables 1 and 2). Bilateral second-order bile duct involvement (i.e., Bismuth classification IV) is no longer classified as T4 in the 8th edition. Other reasons for T4 (e.g., main portal vein involvement) are reclassified as stage IIIb rather than stage IVa. Positive lymph nodes beyond the hepatoduodenal ligament (e.g., aortocaval or celiac nodes) have become M1 disease (stage IVb) rather than N2 disease in the 7th edition. Instead, in the 8th edition, N2 disease (stage IVa) is classified as 4 or more positive regional lymph nodes.

**Table 1:** American Joint Committee on Cancer (AJCC) staging system by tumor–node–metastasis (TNM) stage on imaging

Stage	AJCC, 7th edition	AJCC, 8th edition
Tumor (T) stage		
T1	Tumor confined to the bile duct, with extension up to the muscle layer or fibrous tissue	
T2a	Tumor invades beyond the wall of the bile duct to surrounding adipose tissue	
T2b	Tumor invades adjacent hepatic parenchyma	
T3	Tumor invades unilateral branches of the PV or HA	
T4	Tumor invades main PV or its branches bilaterally, or the common hepatic artery, second-order bile ducts bilaterally, unilateral second-order bile ducts with contralateral portal vein or hepatic artery involvement	Tumor invades main PV or its branches bilaterally, or the common hepatic artery, or unilateral second-order biliary radicals with contralateral portal vein or hepatic artery involvement.
Node (N) stage		
N0	No regional lymph node metastasis	No regional lymph node metastasis
N1	Regional lymph node metastasis: hilar (along CBD, cystic duct, HA, or PV)	One to three positive lymph nodes typically involving the hilar, cystic duct, common bile duct, hepatic artery, posterior pancreatoduodenal, and portal vein lymph nodes
N2	Metastasis to periaortic, pericaval, SMA, or coeliac lymph nodes	Four or more positive lymph nodes from the sites described for N1
Metastasis (M) stage		
M0	No distant metastasis	No distant metastasis
M1	Distant metastasis	Distant metastasis (includes lymph node metastasis distant to the hepatoduodenal ligament)

**Table 2:** American Joint Committee on Cancer (AJCC) staging system

AJCC, 7th edition				AJCC, 8th edition			
Stage	T	N	M	Stage	T	N	M
0	is	0	0	0	is	0	0
I	1	0	0	I	1	0	0
II	2	0	0	II	2a-b	0	0
IIIa	3	0	0	IIIa	3	0	0
IIIb	1–3	1	0	IIIb	4	0	0
–			0	IIIc	Any	1	0
IVa	4	Any	0	IVa	Any	2	0
IVb	Any Any	2 Any	0 1	IVb	Any	Any	1

AJCC staging systems are intended to be applicable to all cancer patients, regardless whether they undergo curative-intent resection, palliative treatment, or best supportive care. As the majority of patients with PHC is not eligible for curative-intent resection, the AJCC staging involves assessment of cross-sectional imaging in most patients, rather than pathological evaluation of resected tumor specimens. Therefore, the aim of this retrospective study was to evaluate the 8th edition of the AJCC staging system for all patients with PHC and compare the prognostic value of the 7th and 8th editions of the AJCC staging system for PHC.

## Materials and Methods

### Study Population and Data Acquisition

All patients with PHC between 2002 and 2014 in Erasmus MC University Medical Center, Rotterdam, the Netherlands, were included. PHC was defined as a mass or malignant-appearing stricture at or near the biliary confluence, arising between the origin of the cystic duct and the segmental bile ducts.^8^ A multidisciplinary team diagnosed PHC based on clinical characteristics, radiological characteristics, endoscopic findings, and follow-up, if histopathological evidence was not available.^9^ Patient and tumor characteristics, clinical parameters, and laboratory results were retrospectively collected from electronic patient records.

Experienced abdominal radiologists revised all imaging from the time of first presentation. Tumor diameter, presence and location of suspicious lymph nodes, presence of distant metastases, and vascular involvement was reassessed. Suspicious lymph nodes were defined as nodes larger than 1.0 cm in short-axis diameter, with central necrosis, an irregular border, or hyper-attenuation compared to liver parenchyma in the portal-venous contrast-enhancement phase.^9,10^ Vascular involvement was defined as tumor contact of at least 180 degrees to the unilateral or main portal vein or hepatic artery.^9^ Tumor–node–metastasis (TNM) stage was determined according to both the 7th and 8th editions of the AJCC staging system (Table 1). TNM stages I and II were combined, since cT1 (stage I) and cT2 (stage 2) cannot be reliably distinguished on imaging.^11^ The Institutional Review Boards of Erasmus MC University Medical Center approved the study, and the need for informed consent was waived.

### Statistical Analyses

Statistical analyses were performed using IBM SPSS Statistics for Windows version 21.0 (IBM Corp., Armonk, NY, USA) and R (a language and environment for statistical computing) version 3.3.3 for Windows (R Foundation for Statistical Computing, Vienna, Austria). Continuous data are reported as mean with standard deviation (SD) or median with interquartile range (IQR). Categorical parameters are reported as counts and percentages. Survival was measured from the date of first presentation. Survival probabilities were estimated using the Kaplan–Meier method and compared with the log-rank test. Survival status was updated using the municipal records database on December 21, 2017.

Comparison of the staging systems was performed using the concordance index (c-index) and Brier score. The concordance index is used to evaluate whether a staging system can correctly discriminate between two patients at different stages of disease. It is calculated as the probability that for two random patients with different stages, the patient at the lower stage has a longer survival. A c-index of 0.5 means that the predictive ability is no better than random chance. A c-index of 0.7 indicates a good model and an c-index of 1 means perfect prediction. The Brier score is used to measure the difference between observed and predicted survival per stage. As opposed to c-indices, a lower Brier score is better and a score of 0 means total accuracy, while a score of 0.250 indicates no prognostic value.

## Results

### Patient Characteristics

**Fig. 1:**
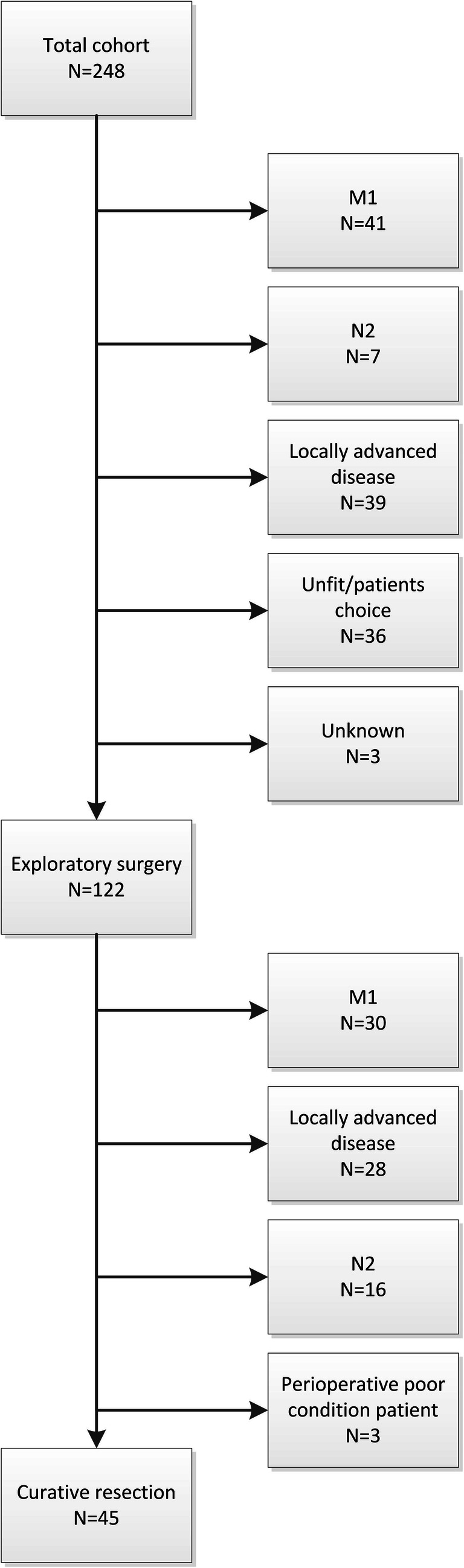
Flow diagram of patient cohort

A total of 248 patients were included; 45 patients (18.1%) underwent a curative-intent resection and 203 patients (81.9%) did not undergo a curative intent resection because they were unfit for surgery or were diagnosed with locally advanced or metastatic disease during workup (Fig. 1). Patient characteristics are summarized in Table 3. Patient characteristics are summarized in Table 3. The median age was 65 years (IQR 55–73) and 150 patients (60.5%) were male. Most patients (*n* = 106, 44.0%) had an ECOG performance status of 0 and 87 patients (35.1%) had a tumor larger than 3 cm on imaging. Unilateral involvement of the portal vein was observed in 87 patients (35.2%) and main/bilateral involvement in 38 (15.4%). Unilateral involvement of the hepatic artery was observed in 107 patients (43.1%) and main/bilateral involvement in 27 (10.9%). The median OS (95% confidence interval (CI)) of the entire cohort was 9.7 months (8.0–11.5).

**Table 3:** Baseline characteristics (*n* = 248)

Characteristic	All patients (*n* = 248)	Curative-intent resection (*n* = 45)	No resection (*n* = 203)
Age at first presentation (years)	65 (55–73)	63 (52–71)	65 (56–73)
Gender, male	150 (60.5)	28 (62.2)	122 (60.1)
Primary sclerosing cholangitis	19 (7.6)	4 (8.9)	15 (7.4)
BMI (kg/m^2^)	24.8 (22.4–27.3)	25.0 (22.1–26.7)	24.8 (24.8–27.5)
ECOG performance status			
0	107 (44.0)	21 (47.7)	85 (43.1)
1	86 (35.4)	14 (31.8)	72 (36.5)
2	37 (15.2)	7 (15.9)	29 (14.7)
3	13 (5.3)	2 (4.5)	11 (5.6)
Jaundice at presentation^a^	192 (80.3)	34 (79.1)	158 (80.6)
CA 19.9 (U/mL)^2^ ≥ 1000 U/mL^b^	46 (27.5)	2 (4.4)	44 (21.7)
Tumor size > 3 cm on imaging	87 (35.2)	7 (15.6)	80 (39.6)
Blumgart stage			
1	71 (29.2)	16 (36.4)	55 (27.6)
2	61 (25.1)	12 (27.3)	49 (24.6)
3	111 (45.7)	16 (36.4)	95 (47.7)
Portal vein involvement^c^			
Unilateral involvement	87 (35.2)	12 (26.7)	75 (37.1)
Main/bilateral involvement	38 (15.4)	2 (4.4)	36 (17.8)
Hepatic artery involvement^c^			
Unilateral involvement	107 (43.1)	15 (33.3)	92 (45.3)
Main/bilateral involvement	27 (10.9	2 (4.4)	25 (12.3)

Categorical parameters are presented as counts (percentages) and continuous parameters as median (interquartile range)

*BMI* body mass index, *ECOG* Eastern Cooperative Oncology Group, *CA 19.9* carbohydrate antigen 19.9;

^a^Missing data for 9 patients

^b^Missing data for 81 patients

^c^Tumor contact of at least 180 degrees to the portal vein or hepatic artery and included main, bilateral, or unilateral involvement on contrast-enhanced CT or MRI imaging

### Staging and Stage Transitions

The 7th edition of the AJCC staging categorized 33 (13.3%) patients in TNM stage I/II, 78 (31.5%) in stage IIIA, 25 (10.1%) in stage IIIB, 41 (16.5%) in stage IVA, and 71 (28.6%) patients in stage IVB. The 8th edition of the AJCC staging categorized 33 (13.3%) patients in stage I/II, 78 (31.5%) in stage IIIA, 11 (4.4%) in stage IIIB, 35 (14.1%) in stage IIIC, 20 (8.1%) in stage IVA, and 71 (28.6%) patients in stage IVB.

Tables 4 and 5 is a cross-tabulation of stage distribution and transitions for the AJCC stages for the 7th and 8th editions. A total of 53 patients (21.4%) were reclassified when considering substages (e.g., stage IIIa and IIIb) and 35 patients (14.1%) considering only the major stages (i.e., stage I, II, III, or IV). Staging according to the 8th edition upstaged 25 patients (10.1%) and downstaged 28 patients (11.3%) of patients in comparison with the 7th edition. Patients with N1 disease (stage IIIb) in the 7th edition were upstaged to IIIc (if 1–3 positive lymph nodes) or IVa (if 4 or more positive lymph nodes) in the 8th edition. Most patients with T4 disease (stage IVa) in the 7th edition were downstaged to IIIb (if node-negative) or IIIc (if 1–3 positive lymph nodes) in the 8th edition.

**Table 4:** Cross-tabulation of the main stages of the 7th and 8th editions of the American Joint Committee on Cancer (AJCC) staging system

		8th edition			
		I/II	III	IV	Total
7th edition	I/II	33	0	0	33
	III	0	96	7	103
	IV	0	**28**	84	112
	Total	33	124	91	248

Each row shows how many patients at a specific 7th edition stage transitioned to other stages according to the 8th edition. Numbers in **red** refer to patients who moved to a different stage from the 7th to the 8th edition

**Table 5:** Cross-tabulation of the substages of the 7th and 8th editions of the American Joint Committee on Cancer (AJCC) staging system

		8th edition						
		I/II	IIIa	IIIb	IIIc	IVa	IVb	Total
7th edition	I/II	33	0	0	0	0	0	33
	IIIa	0	78	0	0	0	0	78
	IIIb	0	0	0	**18**	**7**	0	25
	IVa	0	0	**11**	**17**	13	0	41
	IVb	0	0	0	0	0	71	71
	Total	33	78	11	35	20	71	248

Each row shows how many patients at a specific 7th edition stage transitioned to other stages according to the 8th edition. Numbers in **red** refer to patients who moved to a different stage from the 7th to the 8th edition

### Survival across Stages

**Fig. 2:**
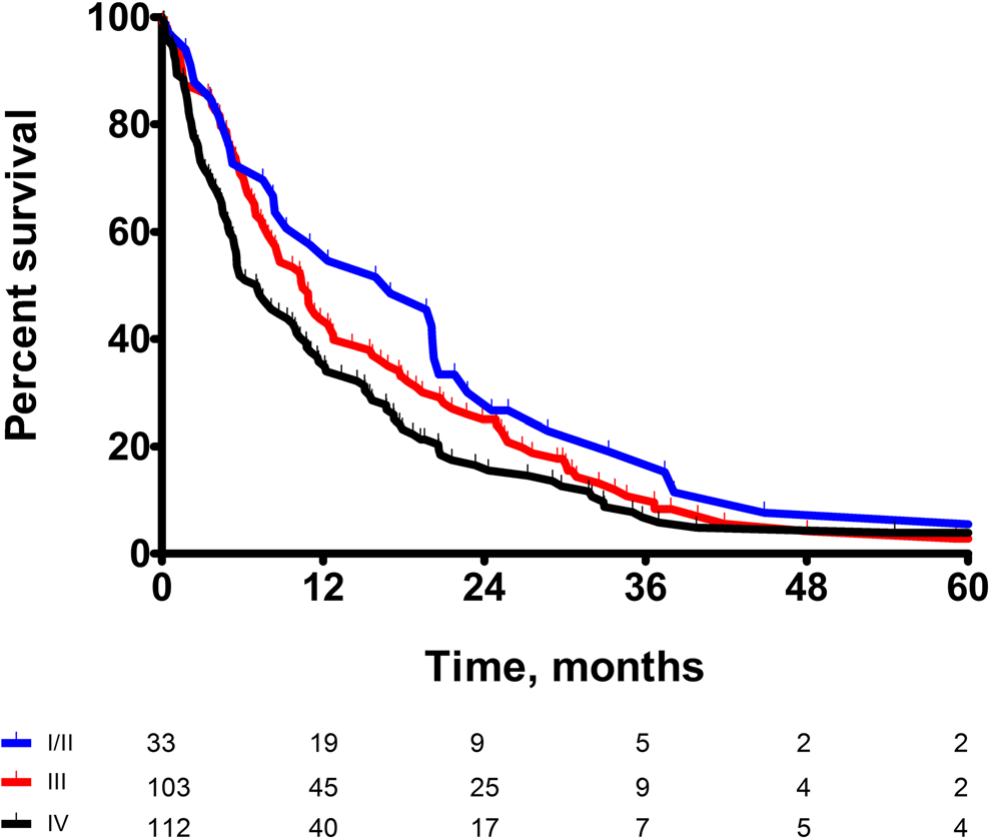
Kaplan–Meier curves for OS for the main stages of the 7th edition

**Fig. 3:**
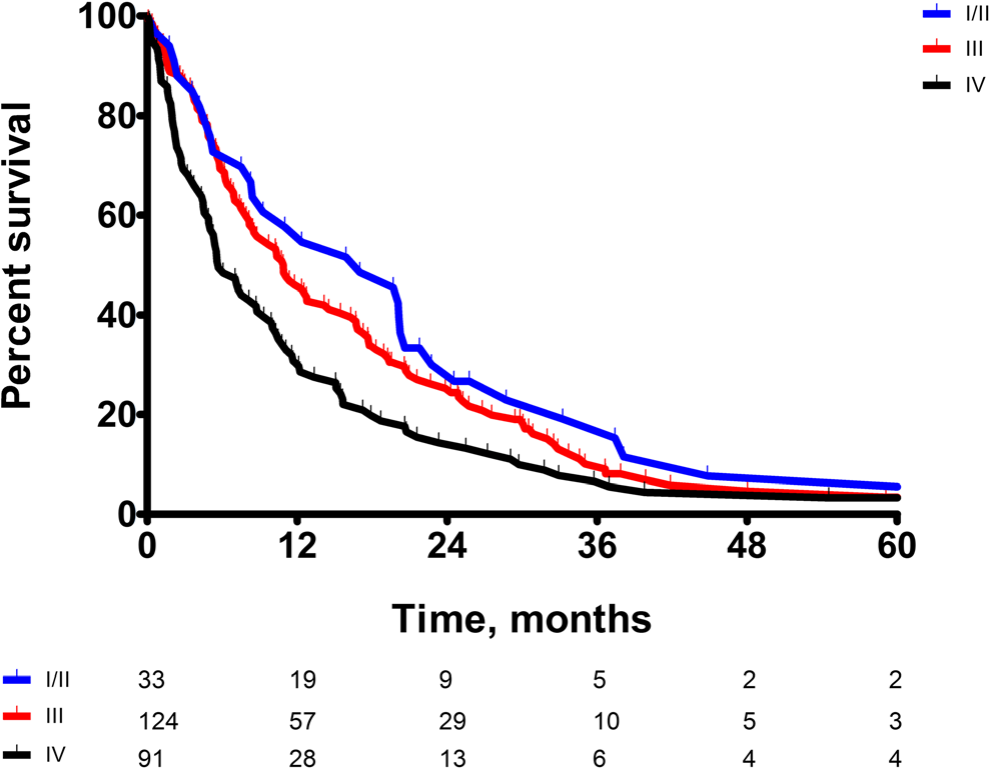
Kaplan–Meier curves for OS for the main stages of the 8th edition

The median OS for patients staged according to the 7th or 8th edition per TNM stage were as follows: stage I/II (17.0 vs. 17.0 months), stage III (10.5 vs. 10.9 months), and stage IV (7.03 vs. 5.6 months), respectively (*p* value between stages in the 7th edition = 0.085 vs. *p* value between stages in the 8th edition = 0.015). Figures 2 and 3 show the Kaplan–Meier curves for OS for the main stages of the 7th and 8th editions.

### Prognostic Accuracy

Table 6 shows the concordance indices and Brier scores for the two editions of the AJCC staging system. Prognostic accuracy of the 8th editions of the main stages of the AJCC staging systems was slightly higher than the 7th edition (c-index 0.59 vs 0.61). Expanding the 7th edition to include substages (e.g., IIIa and IIIb) slightly diminished its prognostic accuracy (c-statistic from 0.59 to 0.57). Expansions of the 8th edition also diminished its prognostic accuracy (c-statistic from 0.61 to 0.58). Prognostic accuracy was comparable between the expanded 7th and 8th AJCC staging systems (c-index 0.57 vs 0.58).

**Table 6:** Predictive accuracy of the various staging systems. A high concordance index in better, a low brier score is better

	Concordance index	Brier score^a^
Entire cohort		
AJCC substages—7th	0.57	0.24
AJCC substages—8th	0.58	0.23
AJCC main stages—7th	0.59	0.24
AJCC main stages—8th	0.61	0.24
Subgroup—curative-intent resection		
AJCC substages—7th	0.65	0.15
AJCC substages—8th	0.67	0.15
AJCC main stages—7th	0.65	0.15
AJCC main stages—8th	0.64	0.15
Subgroup—no resection		
AJCC substages—7th	0.54	0.22
AJCC substages—8th	0.57	0.22
AJCC main stages—7th	0.56	0.23
AJCC main stages—8th	0.57	0.22

^a^Brier score calculated for 1 year for both the total cohort and the no resection subgroup and calculated for 3 years in the curative-intent resection subgroup

Subgroup analysis was performed to determine the prognostic accuracy of the AJCC staging system editions across treatment groups (Table 6). In both the 7th as the 8th editions, the AJCC staging system performed better in the subgroup of patients who underwent a curative-intent resection compared to the entire cohort (0.65 vs 0.57 in the 7th edition, 0.67 vs 0.58 in the 8th edition). The 8th edition did have a slightly better prognostic value compared to the 7th edition in this subgroup (c-index of 0.65 vs 0.67).

Although the prognostic accuracy of the 8th edition of the AJCC staging system in patients who did not undergo a resection was slightly better when compared to the 7th edition (0.54), the prognostic accuracy was still very poor with a c-index of 0.57 in both the main as expanded staging system.

## Discussion

In our all-comer cohort, we found that the prognostic accuracy of cross-sectional imaging for patients presenting with PHC was comparable across the 7th and 8th AJCC staging systems (c-index 0.57 vs 0.58). The prognostic accuracy of the 8th edition was higher in patients who underwent a curative-intent resection compared with those who did not (0.67 and 0.57). Although prognostic accuracy of the 8th edition in patients who did not undergo a curative-intent resection was slightly better than the 7th edition, the prognostic accuracy of the AJCC staging system in these patients was still poor with a c-index of 0.57.

The 8th edition AJCC staging system included four major modifications (Table 1). These modifications resulted in reclassification of 53 (21.4%) patients with consideration of substages (e.g., stages IIIa and IIIb) and 35 (14.1%) patients considering only the major stages. However, these modifications and concomitant reclassifications failed to significantly improve its prognostic accuracy.

Other studies evaluated the prognostic accuracy of the 7th edition of the AJCC staging system.^12–14^ However, TNM stages were based on pathological evaluation (pTNM) of the resected specimen, rather than evaluating cross-sectional imaging (cTNM) as was performed in the present study. These studies excluded most PHC patients, because only a minority of PHC patients is eligible for a curative-intent resection. A large study comparing the 6th and 7th editions of the AJCC staging system in a cohort of 306 patients who underwent a resection found similar prognostic accuracy for the 7th edition with a c-index of 0.59 using only the main stages and 0.54 using substages.^12^ . A Japanese study evaluated the 7th edition of the AJCC staging system and proposed a modified system.^13^ This modification was the basis for the modification in T stage implemented in the 8th edition of the AJCC staging system: Bismuth type IV tumors were no longer considered as T4 and T4 tumors were downstaged from stage IVA to IIIb. However, external validation showed that the modified model did not improve prognostic accuracy compared to the 6th and 7th editions of the AJCC staging system.^12^

This is the first study to evaluate the 8th edition of the AJCC staging system for all patients with PHC, regardless of subsequent treatment. AJCC stages were assigned based on cross-sectional imaging (cTNM). Stage assignment based on pathological evaluation (i.e., pTNM) was not possible, because most patients with PHC have locally advanced or metastatic disease or are unfit to undergo major surgery and therefore do not undergo a resection. Nevertheless, this study has some limitations that should be mentioned. The TNM stage was determined on cross-sectional imaging in all patients with PHC, rather than using pathological examination of resected specimens. Vascular involvement and the biliary extent of the tumor are often difficult to determine on cross-sectional imaging. However, the AJCC staging system is specifically developed to apply on both cross-sectional imaging and pathological examination of all PHC patients. In future studies, we would like to compare clinical and pathological staging, which would require detailed pathological reporting.

Because most patients with PHC have locally advanced or metastatic disease at presentation (or are unfit for major surgery), the prognostic accuracy of AJCC staging system editions should be based on cross-sectional imaging rather than pathological evaluation. In addition, staging has the most potential clinical implications in the preoperative period, where it can still influence the decision whether to try and perform a resection or not. Accuracy on imaging is therefore arguably the most important parameter. Future editions of the AJCC staging system should aim to improve the prognostic accuracy of AJCC staging system on cross-sectional imaging.

## Conclusions

The prognostic accuracy of the 8th edition of the AJCC staging system was similar to the 7th edition. Prognostic accuracy was particularly poor in the majority of PHC patients who did not undergo a resection. Future editions of the AJCC staging system should aim to improve the prognostic accuracy of AJCC staging system on cross-sectional imaging.
